# Implementation of Reference Framework for Preventive Care for Older Adults: Cross-sectional Qualitative Study

**DOI:** 10.2196/78814

**Published:** 2026-03-04

**Authors:** Claire Chenwen Zhong, Mingtao Chen, Chung Yi Lo, Man Kin YIM, Xiaoshu Zhang, William CW Wong, Junjie Huang, Martin CS Wong

**Affiliations:** 1 JC School of Public Health and Primary Care Faculty of Medicine Chinese University of Hong Kong Hong Kong China (Hong Kong); 2 Centre for Health Education and Health Promotion Faculty of Medicine Chinese University of Hong Kong Hong Kong China (Hong Kong); 3 Department of Family Medicine & Primary Care LKS Faculty of Medicine University of Hong Kong Hong Kong China (Hong Kong)

**Keywords:** primary care, guideline adherence, geriatrics, implementation science, Consolidated Framework for Implementation Research

## Abstract

**Background:**

The Hong Kong Government developed the Reference Framework for Preventive Care for Older Adults (RF) to provide evidence-based recommendations for preventive care in the primary care setting. However, no study has systematically evaluated the adoption and awareness of this framework among primary care physicians (PCPs).

**Objective:**

This study aimed to explore PCPs' perceptions of the RF and identify barriers and facilitators to its adoption in primary care settings.

**Methods:**

This cross-sectional qualitative study involved interviews with 40 PCPs in Hong Kong to assess their views on the RF’s adoption and implementation. The Consolidated Framework for Implementation Research (CFIR) was used to guide the analysis, focusing on five domains: intervention characteristics, outer setting, inner setting, individual characteristics, and implementation process.

**Results:**

Relative advantage (65%, n = 26), innovation design (45%, n = 18), and access to knowledge and information (70%, n = 28) were the facilitators that mostly discussed by interviewee. By contrast, interviewees also raised concerns regarding policy and legal (n=29, 73%) and availability of resources (n=29, 73%) in the implementation of the RF. In general, participants acknowledged the RF's evidence-based foundation and comprehensive design, appreciating its potential to improve care for older adults. However, barriers included the complexity and limited personalization of the RF, competing clinical priorities, time constraints, and resource limitations. Government support and policy initiatives facilitated engagement, but clearer integration into existing workflows and stronger promotion were needed. Tailored updates, user feedback, and technological enhancements were seen as essential for improving the RF’s usability and ensuring its relevance in clinical practice.

**Conclusions:**

This study highlights that while the RF has potential to improve preventive care in Hong Kong’s primary care setting, its adoption is constrained by systemic, organizational, and individual barriers. To ensure its successful integration, flexible implementation, institutional support, tailored incentives, and enhanced clinician and patient engagement are essential. These findings offer practical implications for policymakers and healthcare practitioners to refine and promote the RF in routine consultations, and underscore the need for future research to test theory-informed implementation strategies. Furthermore, this study offered novel contributions to the CFIR literatures in systematically investigate physician perspectives on RF for the older adult population within the distinct socio-cultural and structural context of Hong Kong, COVID-19 pandemic impact to the current healthcare system and actionable implementation strategies for Asian populations. Applying the findings from this study, the CFIR-ERIC Matching Tool could be used to address the specific barriers identified in this study and improving implementation outcomes across various healthcare settings.

## Introduction

The Hong Kong population has encountered a serious aging burden, with a higher old-age dependency ratio and old-age population proportion than other East Asian districts such as Macau and Singapore [1]. It has been estimated that the proportion of residents aged 65 years or older will increase from 1.45 million (20.5%) in 2021 to more than 2.74 million (36%) in 2046 [2,3]. Apart from implications for policy and the provision of social services, this increase could exert a substantial influence on the demand for health care services, including a higher population prevalence of multimorbidity [4]. A systematic review reported that multimorbidity has affected 76.5% of Chinese older adults [5], and its disease burden continues to escalate in aging communities [6]. The challenge posed by these observations is compounded by the increasing prevalence of disabilities, functional decline, cognitive impairment, mood problems, and lack of social support among the older adult population [7], imposing a substantial burden on patients, their caregivers, and the health care system.

A significant body of evidence shows that adherence to guideline-directed preventive interventions can be translated into clinical benefits [8,9]. In addition, evidence-based guidelines can improve public health outcomes by informing physicians, allied health professionals, and policy makers to practice and design tailored health programs in the most effective way.

The Primary Healthcare Office of the Department of Health, the Hong Kong Government, has produced a Reference Framework for Preventive Care for Older Adults (hereafter “RF”), dedicated to providing recommendations for evidence-based preventive care for the older adult population in the primary care setting. A Task Force on Conceptual Model and Preventive Protocols (hereafter referred to as the “Task Force”) was established under the Working Group on Primary Care [10]. The Task Force synthesized recommendations based on the latest evidence for adoption in primary care settings in Hong Kong. A Clinical Advisory Group, consisting of academic professors, stakeholders from professional organizations, experts from the primary care sector, and advocacy groups, was assembled to contribute to and support guideline development. One reference framework is dedicated specifically to preventive care for older adults [7]. The Task Force is charged with promulgating, maintaining, and revising the framework content and promoting its adoption by primary care physicians (PCPs). The RF contains a core document accompanied by 6 modules on specific areas, including (1) health assessment; (2) falls in older adults; (3) dental health care; (4) visual impairment; (5) mental health problems; and (6) cognitive impairment. The RF adopts a life-course approach and was developed with reference to 2 existing reference frameworks on diabetes and hypertension [11,12]. The RF also highlights key success factors for implementation, including adaptability to local environments, needs, and structures. It was anticipated that the adoption of the RF would improve patient care through health promotion, disease prevention, and addressing major health risks among older adults [7].

It is crucial to devise and implement strategies that could enhance health promotion, disease prevention, and preservation of functional ability among older adults [10]. Active aging has been recognized as multidimensional, encompassing a low risk of disease, high functional capacity, and active engagement in one’s life [13]. With increasing age, lifestyle factors become more important determinants of active aging. Hence, engagement of older adults in disease-preventive activities is of paramount importance.

PCPs are in a privileged position to act as a first point of contact, providing comprehensive, continuous, and community-oriented care in a coordinated manner. They offer health education, risk assessment, and regular care for common chronic diseases [14]. These roles also include support for family members and caregivers, as well as referral to a specialist and community care when applicable. The effectiveness of disease prevention in preserving functional ability among the older adult population has been supported by a substantial body of evidence. For instance, a systematic review of randomized controlled trials summarizing the impact of interventions aimed at preventing frailty progression showed that group exercise programs and cognitive training could successfully reduce or delay frailty [15]. These preventive programs were also found to exert favorable effects on frailty indicators with good value for money, especially among very frail community-dwelling participants.

Several implementation science models are available. The Consolidated Framework for Implementation Research (CFIR) was particularly well-suited for this study because of its comprehensive and flexible nature, which allows for exploration of multiple factors influencing the adoption and implementation of the RF. Unlike other implementation science models that focus on specific domains or stages of implementation—for instance, the Theoretical Domains Framework, which emphasizes individual behavior change, or the Reach, Effectiveness, Adoption, Implementation, and Maintenance (RE-AIM) framework, which focuses on reach and effectiveness [16,17]—CFIR provides a holistic approach by integrating 5 key domains: Intervention Characteristics, Outer Setting, Inner Setting, Characteristics of Individuals, and the Implementation Process [18]. This breadth enables CFIR to capture the dynamic interplay among the RF’s design, the health care system’s infrastructure, external influences, and individual practitioner behaviors. Its adaptability to varying contexts makes CFIR especially relevant for understanding barriers and facilitators to implementing a complex intervention such as the RF in diverse health care settings [18], where factors including resource constraints, time limitations, and local needs are critical. Thus, CFIR’s ability to identify patterns across these domains provides actionable insights that are essential for improving the RF’s adoption and impact.

To our knowledge, no study has systematically assessed this RF or examined the level of awareness and adoption of the RF among PCPs, as well as perceived facilitators and barriers to its implementation. This study aims to explore the implementation of the RF in Hong Kong’s primary care settings, using the CFIR to identify key facilitators and barriers.

## Methods

### Study Design

The study employed a cross-sectional qualitative design involving in-depth individual interviews with 40 PCPs. A sample size of 10-50 participants has been recommended in previous qualitative studies, or until data saturation is reached [19-22], to ensure a comprehensive understanding of the RF. This study was designed, conducted, and reported in accordance with the Standards for Reporting Qualitative Research (SRQR; see Multimedia Appendix 2) [23].

This study was conducted within a constructivist paradigm, which posits that knowledge is socially constructed through interactions between individuals and shaped by context. To explore the implementation of the RF, we employed a 2-stage qualitative analysis. First, we conducted a thematic analysis to inductively identify patterns and emergent themes from the interview data. Subsequently, we applied a framework analysis guided by the CFIR to systematically organize and interpret the data within a structured implementation science framework. This approach allowed for both inductive exploration and deductive mapping of findings to established constructs.

### Participant Recruitment

Participants were recruited between February and September 2024 through emails or phone calls. Eligible participants were (1) PCPs who provide preventive care to older adults as part of their routine clinical practice and (2) those who had read the core document and relevant modules of the RF before the interviews. PCPs who did not consult older adult patients in routine practice were excluded. No restrictions were placed on years of clinical experience to ensure a broad range of viewpoints. Eight distinct groups of PCPs were invited to share their experiences with the adoption of the RF. These groups included (1) PCPs employed by health maintenance organizations; (2) PCPs in group practices within the private sector but not affiliated with health maintenance organizations; (3) solo practitioners providing care independently in private clinics; (4) general outpatient clinic physicians working in publicly funded clinics; (5) physicians at Family Medicine Specialist Clinics; (6) Fellows of the Hong Kong College of Family Physicians who are actively involved in teaching and provide preventive care for older adults; (7) PCPs serving as referral partners for District Health Centers; and (8) physicians with formal qualifications in gerontology or geriatric medicine recognized by the Medical Council of Hong Kong. A purposive sampling method was employed to recruit participants from the 8 predefined PCP groups, ensuring comprehensive representation across diverse practice settings.

For groups 1-3, participants were recruited through established professional networks under the Hong Kong Medical Association. For groups 4 and 5, we contacted the Chiefs of Service across all 7 Hospital Authority clusters, with whom we have long-standing research and teaching collaborations, to nominate suitable participants. Participants in group 6 were identified in collaboration with the Fellows of the Hong Kong College of Family Physicians. For groups 7 and 8, eligible physicians were selected based on referral network lists published by the Health Bureau of the Hong Kong Special Administrative Region Government.

### Data Collection

All interviews were conducted via Zoom (Zoom Communications). A semistructured interview guide (Multimedia Appendix 1) was used to explore PCPs’ awareness of, attitudes toward, and perceived barriers to and enabling factors for use of the RF. Before formal data collection, the guide and the moderator’s manual were pilot-tested with a small sample of PCPs from diverse practice settings. Feedback from the pilot informed revisions to improve the clarity and contextual relevance of the questions. During the interviews, the final version of the guide was used, with additional probing questions introduced as new topics emerged. All interviews were conducted by 2 trained research assistants (CYL and XZ)—one serving as the facilitator and the other as an observer—both of whom had prior experience in qualitative data collection.

All interviews were audio-recorded and transcribed verbatim in Chinese. A standardized transcription protocol was established to guide and ensure consistency throughout the transcription process. Transcribers received training and were supervised by an experienced researcher, who also validated the transcripts to ensure accuracy and reliability. Transcripts were translated into English only after completion of data analysis to preserve the original meanings and avoid introducing interpretive bias during coding. Interviewee transcript review was not conducted, as evidence from previous studies indicates that this practice may have a limited impact on transcript accuracy [24]. However, each transcript was independently reviewed by an experienced researcher (CCZ) to minimize potential bias and misinterpretation. The research team further reviewed all transcripts to ensure both linguistic and conceptual accuracy.

### Data Analysis

All data were analyzed concurrently and sequentially using NVivo 12 software (QSR International). Data analysis was conducted in 2 phases using thematic analysis based on the original Chinese transcripts. This approach was adopted to avoid potential misinterpretations that could arise from premature translation. Quotations were translated into English only after completion of data analysis to ensure that original meanings were preserved. The accuracy of the translations was subsequently checked and confirmed by a senior researcher (CCZ). In the first phase of analysis, inductive coding was performed to identify key patterns and themes emerging from the data. In the second phase, these preliminary themes were systematically reexamined using the CFIR as a guiding framework. The CFIR comprises 38 constructs across 5 domains—Innovation, Inner Setting, Outer Setting, Individual Characteristics, and Implementation Process—which may act as barriers or facilitators depending on the implementation context [25]. Applying the CFIR enabled a structured, theory-driven analysis of the contextual factors shaping RF implementation.

The coding process was conducted independently by 2 researchers (MC and XZ). Any discrepancies were discussed and resolved through team consensus. When disagreements persisted, a senior researcher with expertise in implementation science was consulted to adjudicate and facilitate consensus. Redundant or overlapping codes were merged to ensure clarity and consistency. To identify the most frequently reported implementation determinants, the number of PCPs who described experiences or views related to each CFIR construct was also calculated. This approach helped highlight the most salient barriers and facilitators across participants.

### Quality Assurance

In this study, interviewers received comprehensive training to ensure competency in conducting interviews, adherence to the CFIR framework, and consistency across interviews. The training covered key areas, including understanding the CFIR framework, interviewing skills, ethical considerations, mock interviews, and cultural sensitivity. In addition, quality control measures were adopted to ensure the accuracy and reliability of the data collection process. A standardized procedure was developed to guide the interview process, including preinterview preparation and interview conduct. Furthermore, senior researchers (CCZ and MCSW) periodically observed interviews to ensure adherence to the protocol and to provide feedback to interviewers.

### Ethical Considerations

The study was reviewed and approved by the Survey and Behavioral Research Ethics Committee, The Chinese University of Hong Kong (approval number SBRE-22-0193). Oral informed consent was obtained from all participants before the interviews. The original informed consent permitted secondary analysis without additional consent. Consent information included details about the study’s purpose, procedures, potential risks, and benefits, as well as participants’ right to withdraw at any time without penalty. All data were deidentified to protect participant privacy and confidentiality. For identifiable information collected during the primary study, strict data protection protocols were followed, including secure storage on password-protected systems accessible only to authorized personnel. No identifiable information of individual participants is presented in this manuscript or the multimedia appendices. As a token of appreciation, each participant received an HK $100 (US $12.79) coupon upon completion of the interview.

## Results

### Characteristics of the Included Participants

A total of 40 PCPs participated in this qualitative study, offering diverse professional backgrounds and experiences. Most participants were male (n=31, 78%), while 9 (23%) were female (Table 1). Nearly half (n=18, 45%) were recruited from public health care settings, and 36 (90%) had more than 16 years of clinical experience; 24 (60%) reported seeing fewer than 40 patients per day. The majority were family physicians (n=31, 78%).

**Table 1:** Demographic characteristics of participants.^a^

Demographic characteristics		Values, n (%)
**Gender**		
	Male	31 (78)
	Female	9 (23)
**Age (years)**		
	35-45	7 (18)
	46-55	24 (60)
	56-65	8 (20)
	≥66	1 (3)
**Practice experience (years)**		
	5-15	4 (10)
	16-25	13 (33)
	26-35	16 (40)
	36-45	7 (18)
**Practice type (public)**		
	General outpatient clinic	15 (38)
	Family medicine specialist clinics	2 (5)
	Specialist outpatient clinics	1 (3)
**Practice type (private)**		
	Solo	13 (33)
	With partners	2 (5)
	Health maintenance organizations	4 (10)
	University	3 (8)
**Specialty**		
	Family medicine	31 (78)
	Internal medicine	2 (5)
	Community medicine	3 (8)
	Psychiatry	1 (3)
	Geriatric medicine	3 (8)
**Practice size (number of patients seen daily)**		
	0-19	15 (38)
	20-39	9 (23)
	40-59	7 (18)
	60-79	7 (18)
	>100	1 (3)
	Missing	1 (3)

^a^Participants were recruited from 8 distinct groups of service providers from February to September 2024 through emails or phone calls.

### Barriers and Facilitators to the Adoption of the RF Using CFIR

**Figure 1:**
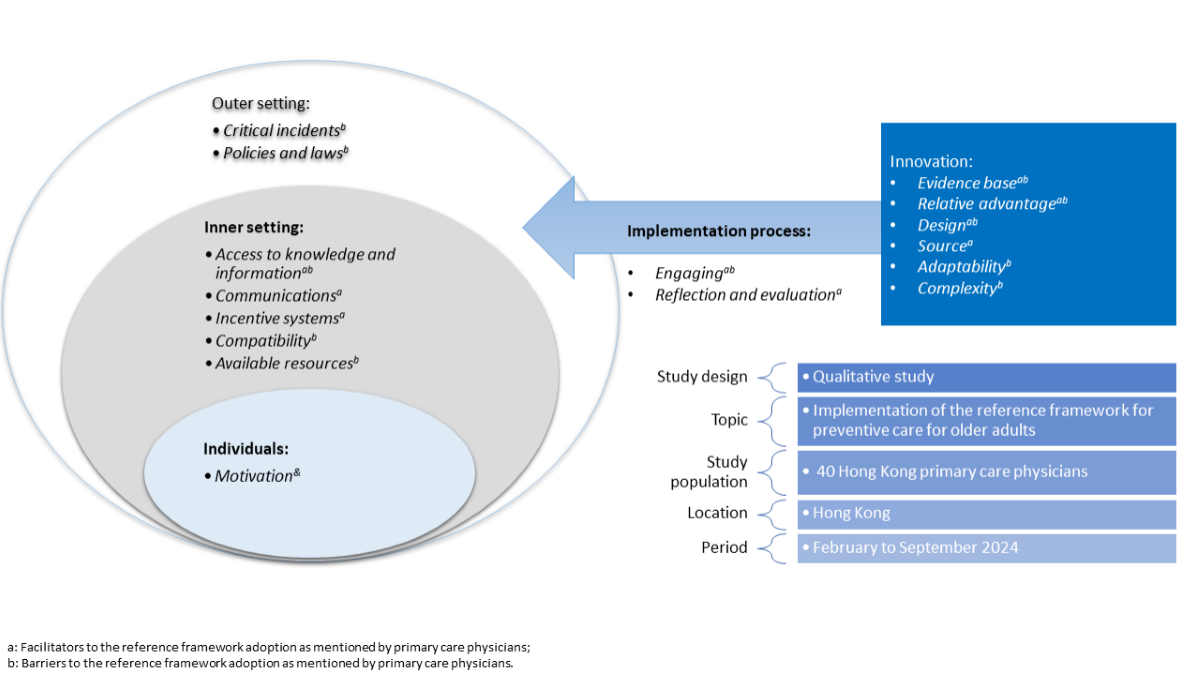
Schematic diagram of the Consolidated Framework for Implementation Research.

Barriers and facilitators to the adoption of the RF in daily consultations were identified using the CFIR framework, with key themes emerging from the interviews. Figure 1 illustrates the CFIR domains reflected in the interviews: Innovation, Outer Setting, Inner Setting, Characteristics of Individuals, and the Implementation Process. Table 2 summarizes the frequencies for each identified theme, while Tables 3 and 4 illustrate quotations for each identified theme. Common facilitators included relative advantage (n=26, 65%), innovation design (n=18, 45%), and access to knowledge and information (n=28, 70%). Major barriers were policy and legal concerns and availability of resources, each cited by 29 (73%) participants. Key themes are detailed below.

**Table 2:** The barriers and facilitators for the reference framework.^a^

CFIR^b^ domains and constructs		Barriers, n (%)	Facilitators, n (%)
**Innovation**			
	Innovation evidence base	4 (10)	7 (18)
	Innovation relative advantage	12 (30)	26 (65)
	Innovation design	10 (25)	18 (45)
	Innovation source	0 (0)	9 (23)
	Innovation adaptability	7 (18)	0 (0)
	Innovation complexity	6 (15)	0 (0)
**Inner Setting**			
	Access to knowledge	10 (25)	28 (70)
	Communications	0 (0)	9 (23)
	Incentive systems	0 (0)	3 (8)
	Compatibility	9 (23)	0 (0)
	Available resources	29 (73)	0 (0)
**Outer Setting**			
	Critical incidents	3 (8)	0 (0)
	Policies and laws	29 (73)	0 (0)
**Individuals**			
	Motivation	3 (8)	0 (0)
**Implementation Process**			
	Engaging	5 (13)	10 (25)
	Reflection and evaluation	0 (0)	12 (30)

^a^Participants were recruited from 8 distinct groups of service providers from February to September 2024 through emails or phone calls.

^b^CFIR: Consolidated Framework for Implementation Research.

**Table 3:** Quotations of facilitators for the reference framework.^a^

CFIR^b^ domains, subdomains, and example categories					Quotations of facilitators
**Innovation**					
	**Evidence base**				
		Confidence in evidence-based approach	“Because it is evidence based, rather than purely based on my own policies for the clinic or purely to meet the needs of patient, it reinforces my beliefs.” (023) “I think that if it will encourage more doctors to, you know, provide evidence-based care, maybe, but it might be worthwhile to sort of know, you know, how much we actually are doing or how successful we are given our current approach” (029) “That is, a lot of evidence base information can actually remind us of some updated treatment plan or some care plan.” (04)		
	**Relative advantage**				
		Value in holistic geriatric care	“We often neglect to consider the living environment of the elderly or whether there is anyone at home to take care of them. This is another important reminder for us—not just to focus on their illness or the medications they are using.” (004) “Dementia is something everyone has heard of, but what exactly is it? What are the symptoms? What should we pay attention to? These are things that, within this framework, help us realize that we need to be aware of these issues, especially for the elderly.” (021)		
		RF^c^ as a structured clinical decision support tool	“It acts as a structured checklist that ensures comprehensive patient assessment.” (009) “A summary checklist facilitates workflow efficiency by allowing clinicians to systematically track completed and pending clinical steps.” (010)		
	**Design**				
		Visual enhancements improve RF usability and implementation	“The color coding is helpful, so you can see things at a glance.” (029) “The hardcopy is of a good size to be put in clinics.” (031)		
		Integration of technology and artificial intelligence to enhance RF implementation	“If we could develop some apps or use AI technology, for example, we could key in certain patient information and come up with conclusions or suggestions. This would be a good direction.” (003) “If there is an advanced version, that is, the RF steps to form a different path, so that the content is more concise, can help me follow the path to care for patients and it will be more convenient for my daily use.” (014)		
	**Source**				
		Trust in the expert-driven development	“You’re talking about doing it based on evidence. Of course, because there’s a large team of experts who spend a lot of time putting it together.” (021) “Having experts provide an official guide for local colleagues is very worthwhile.” (022) “I have confidence in its robustness or feasibility because it is made by the Primary Health Care Office.” (026)		
**Inner Setting**					
	**Access to knowledge and information**				
		Accessibility and availability of RF information facilitate uptake	“Regular updates provided through the Primary Care Office website contribute to sustained engagement with RF.” (011) “Email notifications and printed summary materials enhance accessibility and reinforce RF awareness.” (004)		
	**Communications**				
		Communication and peer support	“Our own doctors’ peer groups have discussions, so from these three aspects, we are aware of this framework.” (005) “Sometimes, there may be specific content that we can discuss together...Maybe we can encourage more to do it.” (010)		
	**Incentive systems**				
		Incentives and Continuing Medical Education accreditation promote RF engagement	“They can also earn some CME credits. This encourages all doctors to reference, learn, and study.” (001) “The government may need to provide more incentives, like funding, to support and motivate doctors to perform these screenings.” (017)		
**Implementation Process**					
	**Engaging**				
		Patient education and shared decision-making facilitate RF integration	“Important points should be highlighted, possibly using charts or visuals to help elderly patients.” (023) “I think the version you give to the patient should not be the English version but the Chinese version...it is a simplified version plus some explanations...we know exactly what we are doing, but for them (the patient), they may think that what you are asking has nothing to do with the disease...Firstly, it needs to be simplified, secondly, it needs some description...Then they (the patients) won't find you annoying and know that what you are asking is really related to them.” (009)		
	**Reflection and evaluation**				
		Continuous evaluation and iterative refinement of RF to ensure relevance	“It would be ideal if there were committees that regularly review this, as that would be very practical.” (015) “I hope the relevant authorities can promptly review the effectiveness of this guideline or framework and propose a post-pandemic version.” (026) “For example...if new decisions arise, do we need to make modifications or release a second edition?...it’s worth paying attention to how often updates occur, or when necessary, updates can be made. (022)		

^a^Participants were recruited from 8 distinct groups of service providers from February to September 2024 through emails or phone calls.

^b^CFIR: Consolidated Framework for Implementation Research.

^c^RF: Reference Framework for Preventive Care for Older Adults.

**Table 4:** Quotations of barriers for the reference framework.^a^

CFIR^b^ domains, subdomains, and example categories				Quotations of facilitators
**Innovation**				
	**Evidence base**			
		Concerns over outdated or overly theoretical content	“The framework is too theoretical, vague academic, and complex, especially for beginners.” (013) “Sometimes I think it's so theoretical, if we can execute the essence of it in front line?” (015) “I also noticed that many of the references in here are from a long time ago, or even 20th century.” (032)	
	**Relative advantage**			
		Limited utility for experienced physicians	“No, it’s not very useful for me. I rely more on what I know than on it...for those who are already trained...it may not be very useful.” (006) “I find it hard to recommend this material. It’s quite simple, and everyone should know it.” (017)	
		Perceived lack of clinical relevance	“The information in the document would not help anyone make significant clinical decisions and is insufficient in determining the next step.” (003) “There might be conditions not currently covered.” (033)	
	**Design**			
		Lack of user-friendliness and accessibility	“It’s not user-friendly because when doctors want to implement certain recommendations, they have to go back to the original documents.” (040) “It almost 70, 80 pages for a module, and not many doctors will read it all...We prefer to read things like lecture notes.” (002) “I’m not sure how much is involved in transitioning to an app version or if there’s a plan for it. Nowadays, everyone accesses information via smartphones.” (034)	
	**Adaptability**			
		Limited personalization and outdated design	“Therefore, I find that what’s mentioned in it is quite vague and not personalized enough to apply to my patients in family medicine.” (006) “After COVID-19, I think there have been significant changes in elderly health...Utilizing technology for smart health care to help the elderly participate in their health has certainly changed compared to the initial design.” (026)	
	**Complexity**			
		Challenges due to breadth and length	“It is also too extensive and should, instead, suggest what choices to consider under different situations.” (014) “It was impractical to follow the guidelines as the RF is too comprehensive with too many pages to follow.” (002)	
**Inner Setting**				
	**Access to knowledge and information**			
		Lack of access to training and awareness	“When we are working, there may be some CME activities on primary care related topics, but we do not pay special attention to attend a workshop on framework.” (022) “I didn’t even know there were any training programs about this” (025)	
	**Compatibility**			
		Compatibility with clinical practice	“Actually, I work in geriatrics. When I talk to patients, the concepts in this overview are quite similar.” (001) “I don't need to go through the overview, because in fact many of them are routines, such as diabetes, hypertension” (014) “We don't usually follow this RF, although sometimes we do things that overlap with the RF, but I don't actually see it from this RF.” (016)	
		Competing priorities and limited focus on the RF^c^	“I haven’t specifically focused on the overview because there are internal workflows and the consultations., we can provide are quite brief due to limited resources.” (014)	
	**Available resources**			
		Time constraints and resource limitations	“In government institutions, like in our general outpatient clinics, it’s quite challenging because we only have 6 minutes for each consultation.” (027) “I look for opportunities during the consultation; if I have extra time, I can discuss a bit more.” (018) “The practicality of the guidelines really depends on how many staff members are available in your clinic and how well you manage your own time.” (007)	
**Outer Setting**				
	**Critical incidents**			
		Disruption caused by COVID-19	“In the past few years, we have been implementing this framework, but many events, like COVID, have occurred.” (019) “I haven't participated in many workshops in the past few years due to the epidemic. Our frontline is mainly responsible for the epidemic.” (023)	
	**Policies and laws**			
		Concerns over compulsory adoption and loss of professional autonomy	“You might have a template for everyone to follow...if someone is entering a nursing home, I need you to fill out all these things, which feels compulsory and may not give the doctor a good impression.” (008) “If it is a policy that we have to follow, then I will have qualified opinion about it...it will make many frontline doctors worry...no more room for freedom...may offend patients, or some patients who have medical needs will not be able to receive suitable treatment.” (004)	
**Characteristics of Individuals**				
	**Motivation**			
		Preference for alternative guidelines	“Some colleagues may follow foreign guidelines instead of the local recommendations.” (040) “Doctors in Hong Kong will refer to a lot of different information...even if I don’t follow Hong Kong’s guideline, but follow US's guideline, it's still evidence base” (035)	
**Implementation Process**				
	**Engaging**			
		Challenges with patient adherence and expectations	“However, when it comes to private practices, if it involves blood tests, it requires citizens to be willing to pay. If a doctor suggests a blood test and the patient is unwilling, that can pose difficulties.” (027) “Currently, the situation is that people come to see a doctor only when they are sick.” (021)	

^a^Participants were recruited from 8 distinct groups of service providers from February to September 2024 through emails or phone calls.

^b^CFIR: Consolidated Framework for Implementation Research.

^c^RF: Reference Framework for Preventive Care for Older Adults.

### Innovation

#### Innovation Evidence Base

##### Confidence in Evidence-Based Approach

The RF’s credibility, grounded in an evidence-based approach, was acknowledged by participants. A total of 7 (18%) respondents mentioned confidence in the use of the RF based on sufficient evidence-based research for its implementation.

Because it is evidence based, rather than purely based on my own policies for the clinic or purely to meet the needs of patient, it reinforces my beliefs.

023

I think that if it will encourage more doctors to, you know, provide evidence-based care, maybe, but it might be worthwhile to sort of know, you know, how much we actually are doing or how successful we are given our current approach.

029

That is, a lot of evidence base information can actually remind us of some updated treatment plan or some care plan.

04

##### Concerns Over Outdated or Overly Theoretical Content

However, the evidence for RF has been subject to some criticism, particularly in terms of its timeliness and clinical validity. Some participants (n=4) indicated that the current evidence for RF was outdated and too theoretical for frontline use.

The framework is too theoretical, vague academic, and complex, especially for beginners.

013

Sometimes I think it's so theoretical, if we can execute the essence of it in front line?

015

I also noticed that many of the references in here are from a long time ago, or even 20th century.

032

#### Innovation Relative Advantage

##### Value in Holistic Geriatric Care

Some participants noted its practical utility. For example, 26 (65%) participants highlighted its value in ensuring no critical aspects are overlooked, particularly in older adults’ care.

We often neglect to consider the living environment of the older adults or whether there is anyone at home to take care of them. This is another important reminder for us—not just to focus on their illness or the medications they are using.

004

Dementia is something everyone has heard of, but what exactly is it? What are the symptoms? What should we pay attention to? These are things that, within this framework, help us realize that we need to be aware of these issues, especially for the older adults.

021

##### RF As a Structured Clinical Decision-Support Tool

In addition, respondents mentioned that this RF helped remind them of some clinical processes that may have been forgotten.

It acts as a structured checklist that ensures comprehensive patient assessment.

009

A summary checklist facilitates workflow efficiency by allowing clinicians to systematically track completed and pending clinical steps.

010

##### Limited Utility for Experienced Physicians

However, 12 (30%) participants pointed out that this RF was not useful to them in practice. On the one hand, they felt that the content of the RF was too basic for experienced physicians.

No, it’s not very useful for me. I rely more on what I know than on it...for those who are already trained...it may not be very useful.

006

I find it hard to recommend this material. It’s quite simple, and everyone should know it.

017

##### Perceived Lack of Clinical Relevance

On the other hand, they believed that the consultation in this RF was insufficient to help them make useful clinical decisions.

The information in the document would not help anyone make significant clinical decisions and is insufficient in determining the next step.

003

There might be conditions not currently covered.

033

#### Innovation Design

##### Visual Enhancements Improve RF Usability and Implementation

Participants (n=18) were optimistic about the design. For example, they claimed that the RF is well-sized and color-coded with visual aids, and that these features improve clarity and accessibility.

The color coding is helpful, so you can see things at a glance.

029

The hardcopy is of a good size to be put in clinics.

031

##### Integration of Technology and Artificial Intelligence to Enhance RF Implementation

In addition, respondents made several suggestions to make the RF more user-friendly. Their suggestions focused on how to make it easier and quicker to access the information they need and use the RF to improve clinical efficiency, emphasizing the integration of emerging technologies, especially artificial intelligence.

If we could develop some apps or use AI technology, for example, we could key in certain patient information and come up with conclusions or suggestions. This would be a good direction.

003

If there is an advanced version, that is, the RF steps to form a different path, so that the content is more concise, can help me follow the path to care for patients and it will be more convenient for my daily use.

014

##### Lack of User-Friendliness and Accessibility

In addition, the design of the RF was criticized by some respondents. Ten participants expressed concerns and dissatisfaction with the current design of the RF, especially in terms of the user-friendliness of interactions and the lack of usability of the currently available version in different situations.

It’s not user-friendly because when doctors want to implement certain recommendations, they have to go back to the original documents.

040

It almost 70, 80 pages for a module, and not many doctors will read it all...We prefer to read things like lecture notes.

002

I’m not sure how much is involved in transitioning to an app version or if there’s a plan for it. Nowadays, everyone accesses information via smartphones.

034

#### Innovation Source: Trust in the Expert-Driven Development

Within this construct, 9 (23%) participants expressed trust in the expert panels involved in developing the RF. They generally believed that the expertise of these panels ensured the reliability of the RF and supported the evidence-based approach adopted.

You’re talking about doing it based on evidence. Of course, because there’s a large team of experts who spend a lot of time putting it together.

021

Having experts provide an official guide for local colleagues is very worthwhile.

022

I have confidence in its robustness or feasibility because it is made by the Primary Health Care Office.

026

#### Innovation Adaptability: Limited Personalization and Outdated Design

Participants (n=7) raised concerns about the local use of this RF at the frontline. They claimed that the lack of personalization or localization in the current version of the RF hinders its use in their practice.

Therefore, I find that what’s mentioned in it is quite vague and not personalized enough to apply to my patients in family medicine.

006

After COVID-19, I think there have been significant changes in older adults’ health...Utilizing technology for smart health care to help the older adults participate in their health has certainly changed compared to the initial design.

026

#### Innovation Complexity: Challenges Due to Breadth and Length

The comprehensiveness of the RF presented a challenge. Some participants (n=6) pointed out that it contained extensive content and too many pages, which limited their ability to implement and follow it in a real-world setting.

It is also too extensive and should, instead, suggest what choices to consider under different situations.

014

It was impractical to follow the guidelines as the RF is too comprehensive with too many pages to follow.

002

### Inner Setting

#### Access to Knowledge and Information

##### Accessibility and Availability of RF Information Facilitate Uptake

Most participants (n=28) indicated that they obtained RF-related information and updates mainly through self-directed behavior or informal pathways, such as checking the websites of government health care departments and emails and hard copies sent by relevant departments, and also considered that this made them more familiar with the RF.

Regular updates provided through the Primary Care Office website contribute to sustained engagement with RF.

011

Email notifications and printed summary materials enhance accessibility and reinforce RF awareness.

004

##### Lack of Access to Training and Awareness

However, some participants (n=10) pointed out that information on RF training was limited and that they had not even received information on RF training or workshops.

When we are working, there may be some CME activities on primary care related topics, but we do not pay special attention to attend a workshop on framework.

022

I didn't even know there were any training programs about this

025

#### Communications: Communications and Peer Support

Maintaining communication with peers is important for encouraging the use of the RF. Nine participants mentioned that they often update information through peer discussions and thus increase peer collaboration, informal feedback on existing content, and review of its utility in the clinic to ensure more effective implementation of the RF.

Our own doctors’ peer groups have discussions, so from these three aspects, we are aware of this framework.

005

Sometimes, there may be specific content that we can discuss together...Maybe we can encourage more to do it.

010

#### Incentive Systems: Incentives and Continuing Medical Education Accreditation

Incentives, tangible or intangible, are crucial to the implementation of new measures. Three participants mentioned that additional incentives may be needed to increase the use of the RF, including the inclusion of the RF in Continuing Medical Education (CME) credit courses to encourage more physicians to participate. In addition, providing financial assistance was cited as an effective approach.

They can also earn some CME credits. This encourages all doctors to reference, learn, and study.

001

The government may need to provide more incentives, like funding, to support and motivate doctors to perform these screenings.

017

#### Compatibility

##### Compatibility With Clinical Practice

Some physicians (n=9) indicated that they were reluctant to use or refer to the RF in their day-to-day work, and one of the primary reasons was that they recognized the content of the RF as similar to what they did on a day-to-day basis.

Actually, I work in geriatrics. When I talk to patients, the concepts in this overview are quite similar.

001

We don't usually follow this RF, although sometimes we do things that overlap with the RF, but I don't actually see it from this RF.

016

I don't need to go through the overview, because in fact many of them are routines, such as diabetes, hypertension.

014

##### Competing Priorities and Limited Focus on the RF

In addition, the prioritization of internal workflows may hinder their use of the RF, as one participant mentioned.

I haven’t specifically focused on the overview because there are internal workflows and the consultations.

014

#### Available Resources: Time Constraints and Resource Limitations

When discussing how the RF works in practice, most participants (n=29) complained that real-world resource constraints limited their use of the RF, especially time constraints. Resource constraints have been a longstanding theme in the health care system, especially in the public health care system, where respondents reported that their consultation time per patient was only a few minutes, making it difficult to implement the RF. In addition, the lack of staff presents an additional challenge.

In government institutions, like in our general outpatient clinics, it’s quite challenging because we only have 6 minutes for each consultation.

027

I look for opportunities during the consultation; if I have extra time, I can discuss a bit more.

018

The practicality of the guidelines really depends on how many staff members are available in your clinic and how well you manage your own time.

007

### Outer Setting

#### Critical Incidents: Disruption Caused by COVID-19

The COVID-19 pandemic posed challenges to the RF’s promotion and implementation, with 3 participants mentioning that the pandemic limited its outreach. They noted that the arrival of COVID-19 shifted their focus to COVID-19–related activities, to the extent that they had little exposure to RF-related knowledge or training, despite having started RF implementation several years ago.

In the past few years, we have been implementing this framework, but many events, like COVID, have occurred.

019

I haven't participated in many workshops in the past few years due to the epidemic. Our frontline is mainly responsible for the epidemic.

023

#### Policies and Laws: Concerns Over Compulsory Adoption and Loss of Professional Autonomy

When we addressed their views on whether the RF should be made a policy, most participants (n=29) showed clear opposition, believing that if the RF is made mandatory, it may cause physicians to lose their clinical freedom, thereby ignoring the real needs of their patients.

If it is a policy that we have to follow, then I will have qualified opinion about it...it will make many frontline doctors worry...no more room for freedom...may offend patients, or some patients who have medical needs will not be able to receive suitable treatment.

004

You might have a template for everyone to follow...if someone is entering a nursing home, I need you to fill out all these things, which feels compulsory and may not give the doctor a good impression.

008

### Individuals (Motivation: Preference for Alternative Guidelines)

We found that PCPs’ beliefs about different health care systems may influence their acceptance and adoption of the RF. Participants (n=3) noted that they may compare practices and guidelines from different regions on their own and place more faith in foreign guidelines than in local recommendations.

Some colleagues may follow foreign guidelines instead of the local recommendations.

040

Doctors in Hong Kong will refer to a lot of different information...even if I don't follow Hong Kong's guideline, but follow US's guideline, it's still evidence base.

035

### Implementation Process

#### Engaging

##### Challenges With Patient Adherence and Expectations

We found that some patients’ uncooperative behaviors or habits may affect physicians’ confidence and willingness to implement the RF. Some participants (n=5) noted that even when the RF is followed to make recommendations for patients, its effectiveness requires patient participation, and that uncooperative attitudes make the RF difficult to implement. In addition, respondents pointed to doubts about the usefulness of the RF, emphasizing that confidence in the RF is low in the current style of patient health service seeking.

However, when it comes to private practices, if it involves blood tests, it requires citizens to be willing to pay. If a doctor suggests a blood test and the patient is unwilling, that can pose difficulties.

027

Currently, the situation is that people come to see a doctor only when they are sick...however, the government’s future direction hopes to detect diseases earlier, intervene sooner, and prevent illnesses from occurring.

021

##### Patient Education and Shared Decision-Making Facilitate RF Integration

Although there are some limitations to RF adoption, some participants (n=10) noted that the RF may be adopted because of the role it plays, particularly in empowering patients and helping doctors and patients reach consensus. Participants noted that uncooperative behaviors may decrease and physician-patient communication may be enhanced if patients are aware of the RF.

I think the version you give to the patient should not be the English version but the Chinese version, and it should be simpler, it is a simplified version plus some explanations...Of course, we know exactly what we are doing, but for them (the patient), they may think that what you are asking has nothing to do with the disease, they are just seeing a doctor...Firstly, it needs to be simplified, secondly, it needs some description...Then they (the patients) won't find you annoying and know that what you are asking is really related to them.

009

Important points should be highlighted, possibly using charts or visuals to help older adults.

023

#### Reflection and Evaluation: Continuous Evaluation and Iterative Refinement of RF to Ensure Relevance

When discussing further promotion of RF adoption, participants (n=12) emphasized the importance of regular review and updating to keep up with the latest trends, especially after experiencing critical incidents such as the COVID-19 pandemic.

It would be ideal if there were committees that regularly review this, as that would be very practical.

015

After COVID-19, I think there have been significant changes in older adults’ health...Utilizing technology for smart health care to help the older adults participate in their health has certainly changed compared to the initial design. I hope the relevant authorities can promptly review the effectiveness of this guideline or framework and propose a post-pandemic version.

026

For example, after five years or a few years, if new decisions arise, do we need to make modifications or release a second edition? I know they would do that, but it’s worth paying attention to how often updates occur, or when necessary, updates can be made.

022

## Discussion

### Summary of Findings

This qualitative study examined the views of 40 PCPs on the adoption of the RF in Hong Kong. The findings revealed a complex interplay of systemic, organizational, and individual-level factors influencing RF implementation. At the policy level, many participants expressed concern about making RF adoption mandatory, fearing a loss of clinical autonomy and flexibility in tailoring care to individual patient needs. Participants preferred the RF as a recommendation rather than a compulsory protocol. Within the Inner Setting, peer discussions emerged as a facilitator of RF awareness and informal learning. However, some physicians perceived the RF as redundant, given its conceptual overlap with their routine practice. Competing clinical priorities and limited consultation time also hindered engagement with the RF. Although most participants accessed RF information through government websites or printed materials, many cited a lack of structured training opportunities and expressed interest in clearer dissemination and CME-accredited courses. Resource constraints—especially time limitations and staffing shortages—posed significant barriers to practical implementation. Furthermore, physicians highlighted the importance of financial and educational incentives to support adoption. At the individual level, some PCPs demonstrated a preference for foreign guidelines over local ones, reflecting skepticism toward the RF’s added value. Patient factors, including low adherence, financial considerations, and a curative (rather than preventive) care-seeking mindset, were seen as major challenges to RF uptake. Despite these barriers, several participants acknowledged the potential of the RF to enhance physician-patient communication and support shared decision-making, particularly if supported by simplified, culturally appropriate patient materials. Continuous evaluation and iterative updates to the RF were viewed as essential to maintaining relevance, especially in the post–COVID-19 context. To further understand how these factors interact, we mapped them across the CFIR domains to identify patterns and relationships that shape implementation outcomes.

### Interpretation Across CFIR Domains

Within the Intervention Characteristics domain, trust in the RF’s evidence-based foundation and expert development fostered credibility, yet its perceived complexity and limited adaptability constrained its practical use—especially in time-constrained environments (Inner Setting). External pressures such as the COVID-19 pandemic and resistance to policy mandates (Outer Setting) further shaped adoption, interacting with individual beliefs and preferences (Characteristics of Individuals), including skepticism toward local guidelines. Peer communication and informal learning (Inner Setting) helped mitigate gaps in formal training, while patient engagement and shared decision-making (Process) emerged as critical to overcoming adherence challenges. These cross-domain patterns suggest that effective implementation requires not only credible and well-designed content but also responsive adaptation to clinical workflows, supportive infrastructure, and active stakeholder involvement.

### Possible Explanation and Recommendations

This study received both positive and negative feedback from practitioners regarding their impressions of the RF, with some practitioners indicating that the RF was useful, while others criticized that the content was not applicable to daily consultation routines. Based on our results, practitioner noncompliance may be related to personal attitudes toward the RF and limitations of the RF, such as too many pages and differing physiological conditions of patients. The results revealed that improvement of the RF was necessary to enhance practitioner compliance. High complexity and a high number of conditional recommendations in guidelines have been found to be major barriers to physicians adopting guidelines [26]. The current RF contains comprehensive information but lacks a quick checklist for practitioners to apply. A simple checklist for practitioners and patients may help improve RF compliance. Furthermore, comments from physicians reflected a lack of collaboration and communication between expert panels and frontline physicians, resulting in a tool that prioritizes general guidance over actionable, context-specific solutions. Lu and colleagues [27] emphasized and provided key strategies for successful implementation, in which stakeholder engagement was identified as an important process for success. To bridge this gap, co-designing updates to the RF with frontline practitioners is essential. Regular feedback loops and workshops involving diverse stakeholders, including PCPs, specialists, and nurses, can help refine the framework. These updates should focus on creating adaptable modules or case-based scenarios that address specific clinical complexities while retaining the RF’s evidence-based rigor. A critical interpretation of our results points to a fundamental implementation challenge: the RF was developed with insufficient input from its end users. This top-down approach likely explains why physicians viewed it as a source of general guidance rather than a practical clinical tool. This aligns with the implementation science literature, which consistently identifies stakeholder engagement as a cornerstone of success [27]. Therefore, a paradigm shift from consultation to co-creation is warranted. We recommend that updates to the RF be developed through participatory workshops with frontline PCPs, specialists, and nurses. This co-design process would focus on embedding actionable, case-based scenarios directly into the framework, enhancing its relevance and utility in daily practice.

The RF received a positive response regarding the user-friendliness and convenient design of the framework, especially in terms of size and color coding. User-friendliness also emerged as a crucial factor in promoting regular use. Catho et al [28] conducted a qualitative study to examine factors determining guideline adherence and adoption of computerized decision support systems by physicians. The study indicated that user-friendliness was one of the key factors affecting the adoption of new tools in the workplace. The underlying mechanism may be attributed to the fact that an intuitive interface and adequate technical support may help clinicians use the RF easily, even if they are not familiar with the technology [29].

By contrast, users also identified barriers to implementing the RF in daily consultations. Resource constraints, especially insufficient consultation time, were reported by most participants and substantially affected RF adoption. A systematic review covering 18 countries and 28 million consultations across 111 publications from 1946 to 2016 revealed that in half of the cases, consultation time was 5 minutes or less [30]. The main contributor to this phenomenon was heavy practitioner workloads, for example, in China [31]. No guideline recommends an optimal consultation time for outpatient clinics, but consultation times of less than 7 minutes were found to be more likely to result in practitioners missing some psychosocial problems that patients have [32], let alone following the RF within such a short consultation. Solo practitioners must negotiate optimal medication for patients, address concerns from patients and families, manage schedule constraints, and—above all—consider patients’, families’, or caregivers’ capacity to follow the recommended regimen within a limited consultation. Despite participants having positive responses to the robustness, feasibility, benefits for patient care, user-friendliness, and convenience features of the RF, they also reflected that sufficient consultation time would be key to whether the RF was practically applicable. Notably, our findings emphasize physicians’ resistance to mandatory use of the RF, possibly due to impairment of job autonomy or negative effects from increased external controls [33].

This study highlights physicians perceived role of patient engagement in the decision-making process of whether to practice the RF and points out that low patient compliance and poor care outcomes may undermine physicians’ confidence and willingness to perform the RF, which may be related to physicians perceived meaning of their work. Previous research has indicated that physicians’ perceptions of health care outcomes and the meaning of their work may influence or prevent the onset of burnout [34], and that there is a negative relationship between burnout and physicians’ perceived value of an innovation or willingness to adopt an innovation [35]. This mechanism reflects the fact that physicians may abandon use of the RF because of perceived lack of patient engagement or ineffective care. Therefore, it may be important to enhance physicians’ sense of meaning and willingness to use innovations by improving patient engagement or adherence. In addition, we found that physicians offered many ideas for improving patient engagement, particularly in the areas of patient empowerment and physician-patient consensus. They noted that the RF should be expanded to include patient education, which would help them better implement the content of the RF. This approach may be effective in that, on the one hand, patient empowerment is evident in stimulating patient engagement and optimizing the use of limited resources such as time, as well as reducing the burden on frontline health care professionals [36,37]. On the other hand, improved physician-patient communication may enhance the likelihood of long-term care outcomes, thereby increasing physician confidence in the use of the RF [34].

Continuous improvement is vital for the sustained relevance and effectiveness of the RF. Regularly seeking user feedback and conducting usability testing could help identify areas for enhancement [38]. Implementing long-term engagement strategies, such as personalized reminders, could also help sustain routine use effectively [39-41]. This study revealed insufficient training or workshops concerning the RF, especially during critical incidents such as COVID-19. To support the adoption of the RF, ongoing training and user support are critical. Workshops, clear instructional materials, and accessible technical support [42] could help practitioners become more familiar with the RF as a whole. By implementing these measures, health care providers could navigate the RF more effectively and better utilize the 6 modules in the RF, improving their overall experience and patient health outcomes.

In this study, some constructs, such as structural characteristics and local attitudes, were not coded or mentioned by physicians. This absence appears to reflect the local context. For example, infrastructure was not perceived as a barrier or facilitator, likely because the RF did not require significant changes to physical or technological systems. Similarly, the limited engagement of patients in the Implementation Process may be attributed to the top-down nature of Hong Kong’s health care system, where patients typically have less access to information and decision-making power compared with health care professionals.

### Implications for Practice and Research

Beyond the specific barriers and facilitators identified, our findings underscore that the broader cultural, organizational, and policy contexts in Hong Kong are not passive settings but active forces shaping RF implementation. Culturally, the longstanding preference for curative care over preventive approaches presents a foundational challenge [43]. This orientation suggests that the success of the RF is not only dependent on its technical integration into clinical workflows but also on a broader societal shift toward preventive health literacy. Without sufficient patient understanding and engagement, even a well-integrated RF may encounter resistance or low adherence in practice. At the organizational level, the structure and pace of the public health care system impose significant constraints. Physicians often operate under intense time pressure, and many clinical workflows are already well established. In this context, any new intervention must demonstrate clear added value and seamless compatibility with existing practices; otherwise, it risks being perceived as redundant or burdensome. Therefore, implementation strategies must be designed with these operational realities in mind, ensuring that the RF enhances rather than disrupts clinical efficiency. From a policy perspective, top-down mandates may be counterproductive if they conflict with the cultural value placed on clinical autonomy and the practical limitations of consultation time. Rather than imposing rigid requirements, policy should play a facilitative role. This includes providing resources to alleviate time constraints and supporting integration of the RF into existing digital infrastructures, such as the eHealth app [44]. Embedding the RF into such platforms could streamline access to patient-specific recommendations, reduce the cognitive and administrative load on practitioners, and ultimately improve adherence.

In addition to digital integration, the development of a lay version of the RF could empower older adults by offering accessible, user-friendly guidance on lifestyle changes and risk awareness. This would not only support patient engagement but also reinforce the preventive orientation that the RF seeks to promote. By equipping patients with the knowledge and tools to participate actively in their care, the RF could become a more effective and sustainable component of routine practice. While financial incentives have been proposed to increase practitioner adherence, previous studies have shown that such incentives often have limited impact on clinical behavior [45-47]. As such, financial incentives should be considered a last resort rather than a primary strategy. Instead, the focus should be on creating a supportive ecosystem in which the RF is perceived as both useful and feasible to implement.

This study offers several novel contributions to the CFIR literature. First, to our knowledge, it is the first to systematically investigate physician perspectives on the RF for the older adult population within the distinct sociocultural and structural context of Hong Kong. Second, our application of the CFIR uncovered specific barriers, such as how the COVID-19 pandemic introduced unique, large-scale disruptions that directly impeded the RF’s promotion and integration. Finally, our findings provide actionable insights for tailoring implementation strategies in similar high-density, public payer–led Asian metropolises, addressing a gap in the current evidence base.

Importantly, future research should build on this foundation by applying the CFIR-Expert Recommendations for Implementing Change (ERIC) Matching Tool, which links CFIR-identified barriers to evidence-based implementation strategies from the ERIC taxonomy [48]. This tool maps 73 strategies to CFIR constructs, supporting the selection of targeted interventions such as preparing champions, conducting educational meetings, and tailoring strategies to local contexts [49]. Empirical studies have demonstrated the utility of this approach in improving implementation outcomes across various health care settings, including primary care and geriatric models [50,51]. Future research should focus on testing CFIR-ERIC–informed strategies to address the specific barriers identified in this study, such as limited leadership engagement, resource constraints, and competing clinical priorities. Comparative studies across different health care systems would help assess the generalizability of these strategies, while mixed methods and longitudinal designs could explore their impact on practitioner compliance, patient outcomes, and system-level sustainability.

### Strengths and Limitations

This study is the first to comprehensively assess the adoption of the RF in the primary care setting, offering new insights and potential policy implications for enhancing clinical adoption of the RF. It draws strength from a substantial sample size of 40 PCPs, whose extensive clinical experience enriched the depth and relevance of the findings. Use of the CFIR provided a structured, theory-informed approach to identifying barriers and facilitators, enhancing the analytical rigor of the study. Moreover, the study contributes to the CFIR literature by applying the framework in a unique sociocultural and health care context, highlighting context-specific determinants that may not be captured in differently structured health systems.

However, several limitations should be acknowledged. First, the study relied on self-reported perceptions, which may introduce bias due to social desirability or recall limitations. Second, the findings are context specific and may not be directly transferable to other health care systems. Comparative studies across different health care systems would help assess the generalizability of these findings. Third, the study focused solely on the perspectives of PCPs, without incorporating views from other stakeholders such as patients, administrators, or policy makers, which may have provided a more holistic understanding of RF adoption. Including perspectives from patients, administrators, and policy makers will be essential to developing a more comprehensive understanding of RF implementation and its long-term viability.

### Conclusions

This is the first study investigating the perception of RF implementation among PCPs in Hong Kong guided by the CFIR, offering essential insights for future enhancements of RF and aiding the translation of evidence into clinical practice. While the RF holds promise for enhancing preventive care and shared decision-making in Hong Kong’s primary care setting, its adoption is currently hindered by systemic, organizational, and individual barriers. To support effective integration into routine practice, flexible implementation, stronger institutional support, tailored incentives, and improved clinician and patient engagement strategies are essential. Policy makers could begin by promoting awareness through targeted advertising and professional workshops, followed by revising and refining RF content to better align with clinical realities and patient needs. Future research should empirically test CFIR-ERIC–informed implementation strategies to address identified barriers, explore patient outcomes to support practitioner engagement, and conduct comparative studies to assess the transferability of RF adoption across diverse health care systems.

## Appendix

Multimedia Appendix 1 Interview guide.

Multimedia Appendix 2 SRQR Checklist.

## Abbreviations

CFIR: Consolidated Framework for Implementation Research
CME: Continuing Medical Education
ERIC: Expert Recommendations for Implementing Change
PCP: primary care physician
RE-AIM: Reach, Effectiveness, Adoption, Implementation, and Maintenance
RF: Reference Framework for Preventive Care for Older Adults
SRQR: Standards for Reporting Qualitative Research

## Data Availability

The data supporting the findings of this study are available from the corresponding author (MCSW) upon reasonable request.
